# White Matter Integrity Deficit Associated with Betel Quid Dependence

**DOI:** 10.3389/fpsyt.2017.00201

**Published:** 2017-10-12

**Authors:** Fulai Yuan, Xueling Zhu, Lingyu Kong, Huaizhen Shen, Weihua Liao, Canhua Jiang

**Affiliations:** ^1^Health Management Center, Xiangya Hospital, Central South University, Changsha, China; ^2^School of Humanities and Social Sciences, National University of Defense Technology, Changsha, China; ^3^Department of Radiology, Xiangya Hospital, Central South University, Changsha, China; ^4^Department of Oral and Maxillofacial Surgery, Xiangya Hospital, Central South University, Changsha, China

**Keywords:** betel quid dependence, diffusion tensor imaging, tract-based spatial statistics, fractional anisotropy, mean diffusivity, anterior thalamic radiation

## Abstract

Betel quid (BQ) is a commonly consumed psychoactive substance, which has been regarded as a human carcinogen. Long-term BQ chewing may cause Diagnostic and Statistical Manual of Mental Disorders-IV dependence symptoms, which can lead to decreased cognitive functions, such as attention and inhibition control. Although betel quid dependence (BQD) individuals have been reported with altered brain structure and function, there is little evidence showing white matter microstructure alternation in BQD individuals. The present study aimed to investigate altered white matter microstructure in BQD individuals using diffusion tensor imaging. Tract-based spatial statistics was used to analyze the data. Compared with healthy controls, BQD individuals exhibited higher mean diffusivity (MD) in anterior thalamic radiation (ATR). Further analysis revealed that the ATR in BQD individuals showed less fractional anisotropy (FA) than that in healthy controls. Correlation analysis showed that both the increase of MD and reduction of FA in BQD individuals were associated with severity of BQ dependence. These results suggested that BQD would disrupt the balance between prefrontal cortex and subcortical areas, causing declined inhibition control.

## Introduction

Betel quid (BQ), also known as betel nut or areca nut, is the fourth most commonly consumed psychoactive substance in the world (1). The World Health Organization regards BQ as a human carcinogen. It has been suggested that long-term BQ chewing can lead to betel quid dependence (BQD) according to the diagnostic criteria of substance dependence in Diagnostic and Statistical Manual of Mental Disorders-IV (DSM-IV), which can lead to decreased cognitive functions, such as attention and inhibition control (2–8). It is estimated that about 600 million people chew various types of BQ worldwide, predominantly in the countries of South and Southeast Asia (9). In Mainland China, BQ chewing is mainly practiced in Hunan and Hainan provinces (10).

Recently, BQD individuals have been reported with altered brain structure and function by use of various neuroimaging techniques (11–18). In the aspect of brain structure, our previous work with voxel-based morphormetry analysis suggested that BQD individuals showed less gray matter volume in dorsolateral prefrontal cortex, insula, ventral medial prefrontal cortex (VMPFC), and orbital frontal cortex (OFC) (18). For the brain function, another work with resting-state fMRI showed BQD individuals had decreased default mode network functional connectivity in VMPFC, OFC, and anterior cingulate cortex (14). Compared with other addictive substance, however, the neural networks and the pathophysiological mechanisms underlying BQ use remain unclear.

To date, there is little evidence examining white matter microstructure alternation in BQD individuals. A recent study using generalized q-sampling imaging reported increased diffusion anisotropy in ACC, parietal, temporal, and occipital regions in BQ chewers compared with healthy controls (12), which indicated that the influence of BQ chewing on the brain white matter. However, relatively small sample size of this study prevented us from drawing solid conclusion on white matter integrity alternation in BQ chewers.

The present study aimed to investigate the differences of white matter integrity between BQD individuals and healthy controls using diffusion tensor imaging (DTI). DTI is a non-invasive technique to study the brain microstructure and the integrity of the anatomical connectivity, which has been applied in studies of many types of addiction, including food (19–21), cocaine (22–24), alcohol (25), gambling (26), internet gaming (27). Fractional anisotropy (FA) and mean diffusivity (MD) are the most common indexes for measuring white matter integrity. Tract-based spatial statistics (TBSS) is a widespread method to process DTI data (28). For example, using TBSS, Romero et al. (22) found that cocaine-dependent subjects presented higher FA values in anterior cingulate and lower FA values in the anterior–posterior commissure plane. Many studies have suggested that there were similar brain mechanism for different types of addiction (29–31). Following this idea, we hypothesized that BQD individuals would show altered white matter compared with healthy controls. We also tried to explore if the FA/MD in certain brain regions could potentially predict the severity of BQ chewing. Understanding the neurobiology feature of BQ chewing would help us develop novel ways to diagnose and prevent BQ dependence.

## Materials and Methods

### Participants

25 BQD individuals were recruited from the outpatient department of stomatology at Xiangya Hospital of Central South University. As reported before (14, 18), BQD individuals were diagnosed by a licensed MD level psychiatrist, according to the Structured Clinical Interview with DSM-IV criteria. Persons without use of BQ were defined as “healthy subjects,” which were recruited through a combination of targeted site sampling, advertisement, and snowball sampling referrals. Participants were excluded if they met criteria for other substance dependence, had a medical history of any neurological or psychiatric disorder, or had claustrophobia/other disease preventing them from MRI scanning. The demographic and clinical characteristics of all participants had been reported in our previous study (18). This study was carried out in accordance with the recommendations of research ethical committee of Xiangya Hospital of Central South University of Hunan Province, Changsha, China. All subjects were given written informed consent in accordance with the Declaration of Helsinki. The protocol was approved by the Institutional Review Board at Xiangya Hospital of Central South University of Hunan Province, Changsha, China.

### Procedures

25 BQD individuals finished the behavior interview. The detailed information on the behavioral interview was reported previously (18). Briefly, they finished the Betel Quid Dependence Scale (BQDS) to test their severity of BQ dependence (32). BQDS is a widely used scale for diagnosing BQD (32), which is a 16-item self-report instrument and gives scores for physical and psychological urgent need, increasing dose, and maladaptive use. The BQDS has an optimal cut-off score of 4, with the optimal sensitivity up to 0.926, the specificity up to 0.977, and the predictive accuracy up to 99.3%. The BQDS exhibits high degrees of reliability and validity in both English-speaking and Chinese-speaking chewers (32, 33). All the BQD and healthy individuals finished the DTI scan.

### DTI Protocol

All MRI images were acquired using a Siemens Skyra 3T scanner at Xiangya Hospital. Participants lay in the supine position on the scanner bed. Foam pads were used to minimize head motion. They were instructed to have a rest but keep their head very still during the DTI scan. The diffusion-tensor data for each subject was acquired using a diffusion-weighted, single-shot, spin-echo, echo-planar imaging sequence parallel to the line of the anterior–posterior commissure. The acquisition parameters were as follows: repetition time = 12,000 ms, echo time = 72.4 ms, matrix = 128 × 128, field of view = 256 × 256, slice thickness = 3 mm, number of excitations = 2, *b*-value = 1,000 s/mm^2^, no gap, 50 axial slices, 64 directions. A dual spin-echo technique combined with bipolar gradients was employed to minimize the geometric distortion induced by eddy currents.

### TBSS Analysis

The DTI data were processed by FMRIB’s Diffusion Toolbox implemented in FSL (http://fsl.fmrib.ox.ac.uk). Diffusion data was corrected for eddy currents and possible head motion. Images were then skull-stripped (34), aligned to MNI space using FNIRT (35, 36), and resampled to 1 mm^3^. FA and MD were reconstructed by fitting a diffusion tensor model at each voxel. The voxelwise statistical analysis of the FA/MD data was carried out using TBSS (28), part of FSL. The mean FA image was created and thinned to create a mean FA skeleton that represented the centers of all tracts common to the group. Each subject’s aligned FA/MD data were then projected onto this skeleton and the resulting data were fed into voxelwise cross-subject statistics. Finally, group differences of the resulting skeletonized FA/MD images were computed using non-parametric permutation methods [Randomize v2.1 in FSL (37)]. The null distribution at each voxel was constructed using 5,000 random permutations of the data. TFCE was used to correct for multiple comparisons across the whole brain. The mean FA/MD value in each significant cluster was then extracted using *fslmeants* for each individual to do correlation analysis with BQDS. Robust regression was used for all correlation to minimize the impact of outliers using robustfit command in the MATLAB Statistics Toolbox.

## Results

Table 1 showed the demographic and clinical characteristics for BQD individuals and healthy controls. The two groups did not differ in terms of age [*t*(48) = 1.06, *p* = 0.29], but the healthy control group was educated for longer time [*t*(48) = −2.47, *p* = 0.017]. In later analysis, years of education was used as a covariate in the model.

**Table 1:** Demographic and clinical characteristics of participants (M ± SD).

	Betel quid (BQ) individuals	Controls	Statistics
Age (years)	29.87 ± 4.71	28.23 ± 5.92	*t*(48) = 1.06, *p* = 0.29
Education (years)	14.39 ± 5.19	17.31 ± 2.87	*t*(48) = −2.47, *p* = 0.017
Betel Quid Dependence Scale	10.87 ± 1.71	–	–
Duration of BQ chewing (years)	12.96 ± 5.05	–	–
Dosage of BQ chewing (g/day)	48.48 ± 17.54	–	–

**Figure 1:**
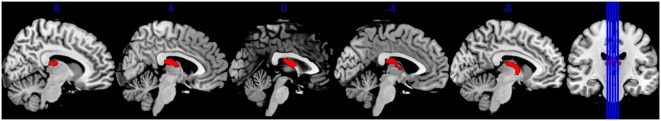
Brain regions showed mean diffusivity difference between Betel quid individuals and controls. Figures are displayed in sagittal view. Numbers above each brain slices were the corresponding × value in the MNI space.

TBSS results showed that, after controlling for education, there was no significant difference in FA between BQD individuals and healthy controls. BQD individuals showed higher MD in bilateral anterior thalamic radiation (ATR) than healthy controls (Table 2). The mean FA values within these two clusters showing MD differences were extracted and compared between these two groups. As showed in Figure 1, results revealed that BQD individuals showed smaller FA values in bilateral ATR than healthy controls [left: *t*(48) = 2.85, *p* < 0.01, *Cohen’s d* = 0.82; right: *t*(48) = 2.93, *p* < 0.01, *Cohen’s d* = 0.85]. Correlation analysis showed that, in BQD group, the FA in bilateral ATR could predict the score in the BQDS [left: *r*(25) = −0.628, *p* < 0.001, right: *r*(25) = −0.637, *p* < 0.001], and the MD in bilateral ATR could also predict the score in the BQDS [left: *r*(25) = 0.529, *p* = 0.006, right: *r*(25) = 0.534, *p* = 0.006].

**Table 2:** Summary of tract-based spatial statistics (TBSS) results (Betel quid individuals > controls).

Brain region		Cluster size	MNI			TFCE-corrected *p*
			*x*	*y*	*z*	
R	ATR	272	3	−5	10	<0.001
L	ATR	153	−6	−3	−1	<0.001

*L, left; R, right; MNI, Montreal Neurological Institute; TFCE, threshold-free cluster enhancement; ATR, anterior thalamic radiation*.

*The voxel size in TBSS analysis is 1 mm × 1 mm × 1 mm = 1 mm^3^*.

## Discussion

Comparing 25 BQD individuals with 25 healthy controls, the present study aimed to investigate altered white matter integrity associated with BQD individuals. Results suggested that BQD individuals showed smaller FA but larger MD in bilateral ATR than healthy controls. Moreover, these measures could potentially predict the severity of BQ dependence.

These results first emphasized the importance of the ATR in BQ dependence. The ATR is the radiation of fibers connecting the anterior and medial thalamic nuclei and the cerebral cortex of the frontal lobe *via* the anterior limb of the internal capsule (38). Altered white matter integrity has been revealed to be associated with major psychiatric disorders in previous investigation [see Ref. (39) for a review], such as depression (40–47), schizophrenia (48), and addiction (49). Especially, studies using DTI in different substance and behavioral addiction have examined addiction-related white matter integrity and reported consistent results in the ATR. For example, Bora and his colleagues found opiate addiction was associated with reduced FA in the ATR, corpus callosum, and longitudinal fasciculus (50). Reduced FA in the ATR and several brain regions was revealed to be related with obesity (21). In pathological gamblers, widespread lower white matter integrity was reported in multiple brain regions including the ATR (26). Furthermore, internet gaming disorder subjects exhibited abnormal FA values in the ATR (27). The pattern of white matter change in the ATR suggested the important role of the ATR in the neurobiology of addiction. Our study extended these prior findings by providing new evidence for abnormal white matter in the ATR in BQD individuals. In addition, we also observed MD increase with BQ dependence in the ATR. MD reflected the average diffusion across three dimensions, the increase of which was suggested to be associated with loss of neurons or myelin. However, few studies suggested MD changes associated with psychiatric disorders (51).

Considering the ATR connects the anterior and medial thalamic nuclei with the prefrontal cortex, the connection between these two neural systems allows us to control the basic impulses and allow more flexible pursuit of long-term goals (19, 52–54). The increased MD and decreased FA within the ATR could be explained as lacking of inhibition from prefrontal cortex to subcortical areas in BQ chewers (53). Similar mechanism has been suggested in both substance and behavioral addiction (19, 52–56), including hyperactivity in subcortical regions and hypoactivity in prefrontal regions (29, 43, 57, 58). Our results further showed that excessive BQ chewing would disrupt the balanced connection between these two neural systems, causing them to enjoy short-term “reinforcing effects” but ignore long-term negative consequences.

It should be noted that there were three limitations in current study. First, the study used a relatively small sample, and we didn’t include female participants, which could potentially limit the generalization of our results to females. Second, the cross-section and correlation methods used in this study may limit our inference on the causality. Further longitudinal study should replicate and extend the conclusion from this study. Third, the conclusion was drawn basically on the structure connectivity, and further studies should try to combine DTI and functional connectivity to confirm our results.

## Ethics Statement

This study was carried out in accordance with the recommendations of research ethical committee of Xiangya Hospital of Central South University of Hunan Province, Changsha, China. All subjects gave written informed consent in accordance with the Declaration of Helsinki. The protocol was approved by the Institutional Review Board at Xiangya Hospital of Central South University of Hunan Province, Changsha, China.

## Author Contributions

FY and XZ wrote the main manuscript text. WL and CJ conceived and designed the experiments. HS and LK conducted the experiments and collected data. FY and LK analyzed the results. All authors reviewed the manuscript.

## Conflict of Interest Statement

The authors declare that the research was conducted in the absence of any commercial or financial relationships that could be construed as a potential conflict of interest.
